# Rapamycin improves endometriosis-related infertility involving ovarian senescence via the PPARα/IGFBP2 pathway

**DOI:** 10.3892/mmr.2025.13722

**Published:** 2025-10-24

**Authors:** Qiongwei Wu, Jiao Fan, Qingjing Sheng, Xiaoying He

**Affiliations:** Shanghai Key Laboratory of Maternal Fetal Medicine, Shanghai Institute of Maternal Fetal Medicine and Gynecologic Oncology, Shanghai First Maternity and Infant Hospital, School of Medicine, Tongji University, Shanghai 200120, P.R. China

**Keywords:** endometriosis, infertility, oxidative stress, senescence, peroxisome proliferator-activated receptor α, insulin-like growth factor-binding protein 2

## Abstract

Endometriosis-associated infertility is considered to be linked to cellular senescence. The present study assessed whether rapamycin, a senescence inhibitor, ameliorates endometriosis-associated infertility by upregulating peroxisome proliferator-activated receptor α (PPARα) and insulin-like growth factor-binding protein 2 (IGFBP2) expression. In the present study, mice were randomized into three groups: Control (CTL), endometriosis (EM) and rapamycin-treated endometriosis (EM-R). The expression of senescence-associated markers at both the tissue and cellular levels were examined as well as the potential mechanisms underlying the effects of rapamycin treatment using ELSA and quantitative PCR. Oxidative stress markers (malondialdehyde and 8-hydroxy-2′-deoxyguanosine) in peritoneal fluid were significantly elevated in the EM group compared with in the EM-R and CTL groups, whilst antioxidant levels (superoxide dismutase and glutathione peroxidase) were lowest in the EM group. Senescence markers (p16, p21 and γH2AX) and gonadotropin receptors (follicle-stimulating hormone receptor and luteinizing hormone receptor) were highest and lowest, respectively, in the EM group. Ovarian analysis revealed a higher primordial follicle count but fewer mature follicles in the EM group compared with the EM-R and CTL groups. Rapamycin treatment increased PPARα and IGFBP2 expression in ovarian tissues, suggesting that its therapeutic effect on endometriosis-related infertility may involve the PPARα/IGFBP2 pathway by mitigating cellular senescence. Rapamycin may potentially ameliorate follicular development in patients with endometriosis.

## Introduction

Endometriosis is a frequent benign gynecological disease that occurs in 10–15% of women of childbearing age. It is typically characterized by endometrial cells and stroma implanting and growing outside the endometrial cavity (1). Endometriosis has been associated with ~50% of women with infertility receiving assisted reproductive technology (2,3). Moreover, women with endometriosis treated with *in vitro* fertilization (IVF) have a lower pregnancy rate and a higher miscarriage rate relative to those without the condition (4,5).

The pathogenesis of endometriosis is not well understood, but several hypotheses have been proposed, including retrograde menstruation, hematogenous/lymphatic dissemination of endometrial cells, coelomic metaplasia, migration of endometrial stem cells, epigenetic modifications promoting inflammation and endocrine disruption due to environmental toxins (6). A key feature of endometriosis is elevated reactive oxygen species (ROS) levels in the pelvic cavity, coupled with reduced antioxidant defenses, suggesting that oxidative stress serves a critical role in disease progression (7). ROS, primarily generated as byproducts of mitochondrial metabolism (8), contributes to cellular senescence, which is a state of irreversible cell cycle arrest accompanied by active metabolic activity and the secretion of senescence-associated secretory phenotypes (SASP). Emerging evidence, including our unpublished data and other studies (9,10), has indicated that cellular senescence is notably associated with endometriotic lesions (9). Furthermore, previous research using cellular and murine models reported that excessive ROS-induced senescence in ovarian granulosa cells contributed to endometriosis-associated infertility (10). However, the precise molecular mechanisms by which peritoneal ROS impair folliculogenesis remain elusive, largely due to ethical constraints limiting functional ovarian studies in young women with endometriosis.

Given that peritoneal fluid surrounds the ovaries (11), we hypothesized that excessive ROS in the pelvic cavity induces ovarian senescence, disrupting follicular maturation and leading to infertility. A hallmark of cellular senescence is the activation of the pro-growth signaling pathway, mammalian target of rapamycin (mTOR), in non-proliferating cells (12). Rapamycin, as an mTOR inhibitor, has been theorized to inhibit cellular senescence, suppress SASP and prolong lifespan (13). Supporting this, a recent clinical study demonstrated that rapamycin administration prior to IVF in women with endometriosis reduced senescence markers in follicular fluid and markedly improved pregnancy and live birth rates (14).

A growing body of evidence has also reported an association between proliferator-activated receptor α (PPARα) and cellular senescence. During aging, PPARα expression or activity was reported to decrease in tissues such as the kidney, heart and spleen (15–17). Insulin-like growth factor-binding protein 2 (IGFBP-2), a target protein of PPARα (18,19), is dynamically regulated in follicular fluid during follicular growth and maturation (20). Notably, IGFBP-2 expression was reported to be reduced in granulosa cells from polycystic ovaries (21) and was upregulated in response to gonadotropin stimulation during final oocyte maturation (22).

Therefore, the present study aimed to assess whether downregulation of the PPARα/IGFBP2 pathway in senescent ovarian tissue impairs follicular development, and whether rapamycin can restore fertility in endometriosis by modulating this signaling axis.

## Materials and methods

### Animals

A total of 50 8-week-old female 18–22 g BALB/c mice were purchased from Shanghai JST Laboratory Animal Co., Ltd., of which 20 were recipient mice, 20 were donor mice and 10 were control mice. All mice were subjected to controlled conditions under a 12 h light/dark cycle with a temperature of 22±2°C and relative humidity of 50±10%, as well as free access to food and water. All experiments were performed under the guidelines of the National Research Council Guide for the Care and Use of Laboratory Animals and were approved by the Institutional Laboratory Animal Review Board of Shanghai First Maternity and Infant Hospital (Shanghai, China; approval no. TJBG28625103).

### Induction of endometriosis and rapamycin treatment

A mouse model of endometriosis was established by intraperitoneal injection of endometrial debris (23). Briefly, after 1 week of adaptation, 20 donor mice were injected intramuscularly with 100 mg/kg estradiol 2–3 times per week (Hangzhou Animal Pharmaceutical (Hangzhou) Co., Ltd.). Euthanasia was performed after 1 week by cervical dislocation. The uteri were removed and collected, and the uterine tissue was cut up and injected intraperitoneally into the abdominal cavity of the recipient mice. The uterine horns of one donor mouse were injected into one recipient mouse. A total of 30 mice (20 recipient mice and 10 control mice) were randomly divided into three groups: i) Control group (CTL group), in which 10 mice received an intraperitoneal injection of saline in equal amounts to the mice in the established mouse endometriosis groups; ii) endometriosis with rapamycin treatment group (EM-R group), in which 10 mice received an intraperitoneal injection of endometrial debris and 250 µg rapamycin/mouse once per week at one 1 after endometriosis induction; and iii) endometriosis group (EM group), in which 10 mice received an intraperitoneal injection of endometrial debris only. Rapamycin was purchased from Sigma-Aldrich (Merck KGaA; cat. no. 553210). The present study referred to the study by Ren *et al* (24) for the dose of rapamycin used in mice. Body weight was measured and the hotplate test was performed in all mice at 1, 2, 3 and 4 weeks after endometriosis induction, and mice were executed 4 weeks after endometriosis induction by cervical dislocation.

### Hotplate test

The hotplate test was performed using a commercial hotplate analgesia meter (BME-480; Institute of Biomedical Engineering, Chinese Academy of Medical Sciences) to assess the degree of discomfort caused by endometriosis, as previously described (25). In summary, mice were given 10 min to acclimate before the test. The mouse had to hop on the hotplate or shake or lick its rear paws during 1 min of being placed within the cylinder to assess the withdrawal latencies to thermal stimulation. Over the course of 1 h, the latency was measured twice and then averaged by the two test results.

### Peritoneal fluid collection and ROS detection in mice

After the mice were executed, they were placed in a supine position with their limbs unfolded and fixed on a dissecting board. The abdomen was disinfected with 75% alcohol, the lower abdominal skin was lifted with ophthalmic forceps and a 2-cm incision was made along the abdominal midline with scissors. A total of 3–5 ml of sterile saline was injected into the abdominal cavity of the mice and the abdominal cavity of the mice was opened under aseptic conditions. The abdominal fluid was rinsed three times and the rinsed fluid was collected into centrifuge tubes for the detection of oxidative stress-related substances. Superoxide dismutase (SOD), malondialdehyde (MDA), glutathione peroxidase (GSH-PX) and 8-hydroxydeoxyguanosine (8-OHdG) were detected as the oxidative and antioxidant markers in the peritoneal fluid of mice. The SOD assay kit (cat. no. A001-3-2; WST-1 method), MDA assay kit (cat. no. A003-1-2; TBA method), GSH-PX assay kit (cat. no. A005-1-2; colorimetric method) and mouse 8-OHdG ELISA kit (cat. no. H165-1-2) were purchased from Nanjing Jiancheng Bioengineering Institute. The peritoneal fluid of PPARα and IGFBP2 in mice were detected using an ELISA kit (cat. nos. MM-0249M1 and MM-0029H2, respectively; Meimian Industry; Jiangsu ELISA Industrial Co., Ltd.). All operations are strictly in accordance with the kit instructions.

### Immunohistochemistry of mouse ovarian tissue

Ovarian tissue samples were fixed in 4% paraformaldehyde for 24 h at 4°C and embedded in paraffin. Sections (4 µm) were used for subsequent staining. The first section was stained with hematoxylin and eosin to confirm the pathological diagnosis, and subsequent sections were stained with senescence-related markers such as p16, p21 and Lamin B1. Tissue sections were deparaffinized in xylene and rehydrated through a graded ethanol series. Antigen retrieval was performed in EDTA buffer (pH 9.0; Shanghai Sun Biotechnology Co., Ltd.) or citric acid solution (pH 6.0; Shanghai Sun Biotechnology Co., Ltd.) using a microwave heating method, then allowed to cool to room temperature. Following antigen retrieval and cooling, sections were washed three times with PBS. To block non-specific binding, sections were incubated in a solution of PBS containing 5% normal goat serum (cat. no. G1208; Wuhan Servicebio Technology Co., Ltd.) for 1 h at room temperature. The following antibodies were used in this experiment: p16 (1:50; cat. no. ab51243), p21 (1:1,000; cat. no. ab188224) and Lamin B1 (1:1,000; cat. no. ab16048). All antibodies were purchased from Abcam. All sections were incubated with the aforementioned primary antibodies overnight at 4°C. The sections were then incubated with horseradish peroxidase-labeled secondary antibody detection reagent (1:300; cat. no. A0303; Beyotime Biotechnology) for 1 h at room temperature. The bound antibody complexes were stained with diaminobenzidine for 3–5 min at room temperature, followed by hematoxylin for nuclear staining at room temperature. The development time was monitored under a microscope (typically between 3 to 5 min) and stopped immediately when optimal staining intensity was achieved with minimal background. Images were captured under a microscope (BX51; Olympus Corporation) equipped with a digital camera (DP70; Olympus Corporation). According to the manufacturer's instructions, distinct tissue slides were utilized for several antibodies as positive control. For negative controls, tissue samples were treated with mouse or rabbit serum (Wuhan Servicebio Technology Co., Ltd.) *in lieu* of primary antibodies.

### Mouse ovarian tissue PCR assay

Mouse ovarian tissues were cut into small pieces (soybean size), placed in a pre-cooled homogenizer, 1 ml RNA extract (Wuhan Servicebio Technology Co., Ltd.) was added per 100 mg of tissue, and the tissues were homogenized until no visible pieces were present. A total of 200 µl chloroform was added per 1 ml RNA extract, and the mixture was vortexed and mixed or shaken upside down vigorously for 15 sed, let stand at room temperature for 3–5 min, then centrifuged at 12,000 × g for 15 min at 4°C. The upper colorless aqueous phase was carefully transferred to a new centrifuge tube, aspirating 500–550 µl per ml of extract. A total of 500 µl isopropanol was added to the collected aqueous extract tube, which was centrifuged at 4°C for 10 min at 12,000 × g. The white precipitate visible at the bottom of the tube was the total RNA, and so supernatant was discarded. A total of 1 ml of 75% ethanol was then added, which was then mixed upside down (until the white precipitate floats) and centrifuged at 12,000 × g for 5 min at 4°C. The supernatant was discarded, then centrifugation was performed briefly at high speed (3–5 sec at 5,000 × g at 4°C) and the liquid was carefully aspirated with a micropipette. The centrifuge tube with RNA precipitate was left open for 3–5 min to allow the RNA to dry slightly, and then 20–50 µl DEPC-treated water (Wuhan Servicebio Technology Co., Ltd.) was added to fully dissolve the RNA. The NanoDrop^™^ Microvolume UV–Vis spectrophotometer (Thermo Fisher Scientific, Inc.) was used to assess the quality control and quantification of total RNA. RNA with good integrity and purity (OD260/OD280 values of 1.8–2.0) was used for further analysis. Mouse tissue RNA was reverse transcribed and quantitative PCR (qPCR) was performed using forward and reverse primers. The cDNA synthesis was performed using a reverse transcription kit (Tiangen Biotech Co., Ltd.) at 42°C for 30 min. To evaluate the abundance of mRNA, real-time PCR was conducted using SYBR Premix Ex Taq (Takara Bio, Inc.). The thermocycling protocol consisted of an initial denaturation step at 95°C for 30 sec, followed by 40 cycles of 95°C for 5 sec and 60°C for 30 sec. GAPDH was used as a housekeeping gene. The sequences of senescence-related markers (p16, p21 and γH2AX), gonadotropin receptor [follicle-stimulating hormone receptor (FSHR) and luteinizing hormone receptor (LHR)] and PPARα and IGFBP2 primers are listed in Table I. All primers were designed by Sangon Biotech (Shanghai) Co., Ltd. The relative gene expression levels were calculated as ΔCq values (Cq ‘target gene’ minus Cq ‘housekeeping gene’) and 2^−ΔΔCq^ ratios indicated fold changes (26).

### Ovarian tissue follicle counting in mice

A total of 30 mice were sacrificed 4 weeks after endometriosis induction. After fixation with 4% paraformaldehyde in phosphate buffer at 4°C for 24 h, the tissues were embedded in paraffin and sectioned at 4 µm thickness. Ovarian tissue sections were stained with HE at room temperature and the number of follicles at different stages was counted for each mouse. The method of follicle counting involved 5-µm continuous sections, with 1/5 sections stained and counted (27). A total of five types of follicles were counted: i) Primordial follicles; ii) primary follicles; iii) secondary follicles; iv) antral follicles; and v) corpus luteum. Primordial follicles were defined as oocytes surrounded by a layer of squamous (flattened) granulosa cells; primary follicles were defined as one follicle containing several or all oocytes surrounded by a single layer of cuboidal granulosa cells; secondary follicles were defined as a follicle surrounded by >1 layer of cuboidal granulosa cells with no visible lumen; a follicle was identified as a antral follicle if it had a well-defined lumen and a layer of cumulus granulosa cells; and the corpus luteum was a post-ovulatory structure, which was filled with lutein cells that form only after ovulation (28). Images were captured using a microscope (BX51; Olympus Corporation) fitted with a digital camera (DP70; Olympus Corporation).

### Granulosa cell culture

Mouse primary granulosa cells were extracted and cultured with reference to the study by Tian *et al* (29). Mouse ovaries were washed with sterile PBS and transferred to DMEM/F12 (cat. no. 11330-057; Invitrogen^™^; Thermo Fisher Scientific, Inc.). Follicles were punctured with a 31-gauge needle under a dissecting microscope, and granulosa cells were released from the follicles, centrifuged at 1,000 × g for 5 min at room temperature, resuspended in fresh DMEM/F12 with 10% FBS, and incubated at 37°C and 5% CO_2_. Cells were passaged every other day.

The granulosa KGN cell line was purchased from Shanghai Kanglang Biotechnology Co., Ltd. and cultured in DMEM/F12 supplemented with 10% FBS at 37°C and 5% CO_2_. Cells were passaged every 5–6 days, and the number of passages was <15 for the experiments.

### Transient transfection for knockdown and overexpression of PPARα

Small interfering (si)RNA of the human PPARα gene (siRNA-PPARα) and non-specific control siRNA (siRNAc) were purchased from Shanghai GenePharma Co., Ltd. and were transfected into cells at room temperature using Lipofectamine^™^ 3000 (Invitrogen; Thermo Fisher Scientific, Inc.) for 48 h at a final concentration of 30 nM, according to the manufacturer's instructions. Subsequent experiments were conducted 48 h after cell transfection. The following siRNA sequences were used: siRNA-PPARα, 5′-GCUUUACGGAAUACCAGUAUU-3′; and siRNAc, (forward) 5′-UUCUCCGAACGUGUCACGUdTdT-3′ and (reverse) 5′-ACGUGACACGUUCGGAGAAdTdT-3′.

The PPARα overexpression plasmid [pCDNA3.1(+) plasmid vector Asian Vector Biotechnology Co., Ltd.] was transfected into granulocyte cells for 48 h using Lipofectamine 3000 and an empty plasmid (Asian Vector Biotechnology Co., Ltd.) was used as a negative control. Cells were transfected with 1.0 µg of either the PPARα overexpression plasmid or the empty control plasmid using 2.0 µl of Lipofectamine 3000 reagent per well in a 12-well plate, according to the manufacturer's instructions. Subsequent experiments were conducted 48 h after cell transfection.

To demonstrated the effects of PPARα knockdown or overexpression on cellular senescence, cells were treated with H_2_O_2_ following knockdown or overexpression. To demonstrated the anti-senescence effects of rapamycin, cells were treated with (50 nM) rapamycin (Sangon Biotech Co., Ltd.).

### Statistical analysis

All statistical analyses were performed using GraphPad Prism 8 (Dotmatics). For comparisons among ≥3 groups, one-way analysis of variance (ANOVA) was employed, followed by Tukey's post hoc test for multiple comparisons when ANOVA indicated statistical significance (P<0.05). Unless otherwise stated, continuous variables are presented as mean ± standard deviation for normally distributed data. All tests were two-tailed, and P<0.05 was considered to indicate a statistically significant difference.

## Results

### Rapamycin reduces ectopic lesions

**Figure 1.**
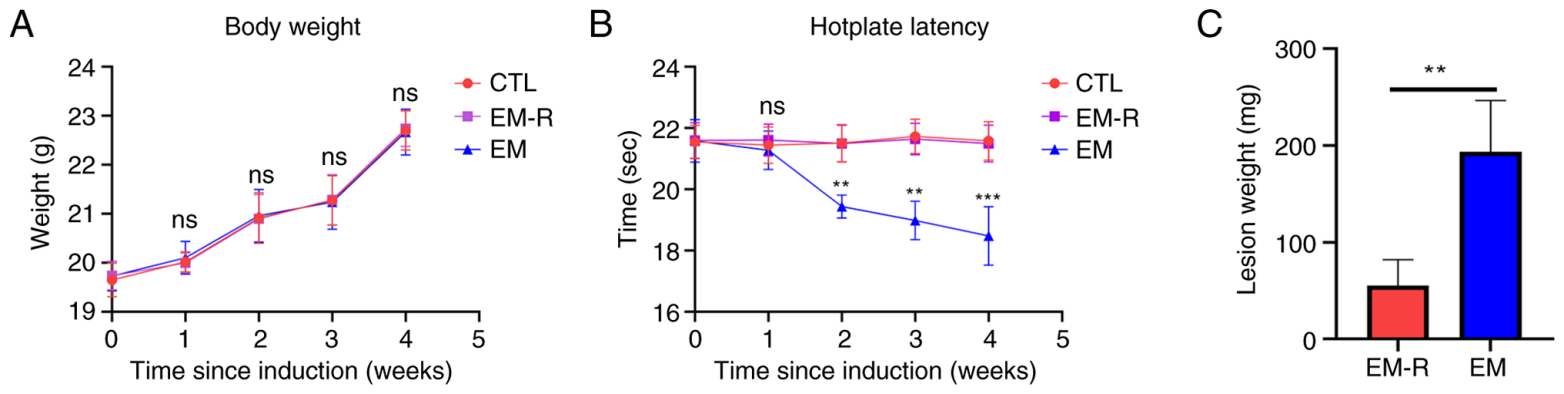
Comparison of mouse body weight, results of the hotplate test and lesion weight. (A) Changes in body weight in the three groups of mice before and after endometriosis induction. (B) Results of the hotplate test in the three groups of mice before and after endometriosis induction. (C) Comparison of lesion weight between the EM and EM-R groups. **P<0.01; ***P<0.001. CTL, control group; EM-R, endometriosis + rapamycin group; EM, endometriosis group; ns, not statistically significant.

The findings demonstrated that there was no significant difference in body weight among the 3 groups of mice before the induction of endometriosis and their death (Fig. 1A). However, the results of the hotplate test were significantly different between the EM group and EM-R group, and the hotplate time from week 2 post-endometriosis induction was significantly lower in the mice of the EM group than in the EM-R group. There is no difference in hotplate test between CTL group and EM-R group (Fig. 1B). No lesions of endometriosis were found in the mice of the CTL group; however, obvious ectopic lesions were observed in the peritoneal cavity of mice in the EM-R and EM groups. Moreover, the weight of endometriotic lesions in mice in the EM group were significantly higher than that in the EM-R group (193.4±53.22 and 55.4±26.69 mg, respectively; P<0.01; Fig. 1C). This indicates that rapamycin may inhibit endometriosis.

### Rapamycin decreases expression of markers associated with oxidative stress in mouse peritoneal fluid

**Figure 2.**
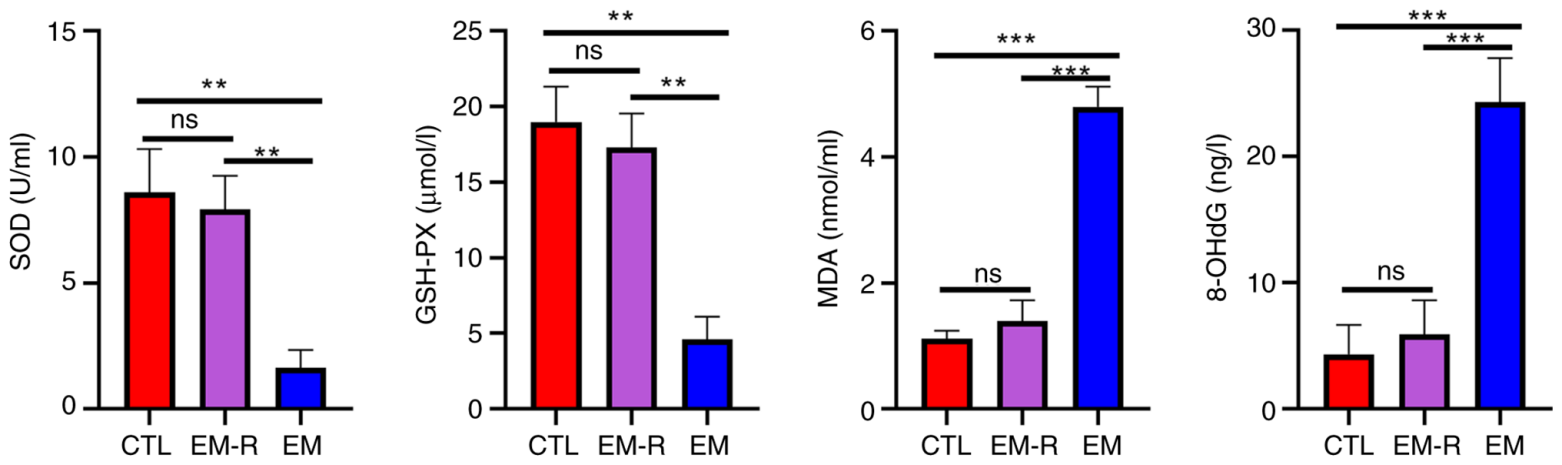
Detection of oxidative stress-related markers in peritoneal fluid of mice. Comparison of antioxidant-related molecules, SOD, GSH-PX and oxidative capacity-related molecules MDA and 8-OHdG in peritoneal fluid of three groups. **P<0.01; ***P<0.001. SOD, superoxide dismutase; GSH-PX, glutathione peroxidase; MDA, malondialdehyde; 8-OHdG, 8-hydroxydeoxyguanosine; CTL, control group; EM-R, endometriosis + rapamycin group; EM, endometriosis group; ns, not statistically significant.

There was no significant difference between the CTL and EM-R groups for the levels of oxidative stress-related molecules, SOD (8.60±1.71 and 7.93±1.32 U/ml, respectively; P=0.49), GSH-PX (18.99±2.32 and 17.30±2.25 µmol/l, respectively; P=0.17), MDA (1.11±0.13 and 1.39±0.32 nmol/ml, respectively; P=0.06) and 8-OHdG (4.34±2.31 and 5.91±2.68 ng/l, respectively; P=0.45). The antioxidant molecules, SOD and GSH-PX, in mouse peritoneal fluid were significantly lower in the EM group compared with in the EM-R group (SOD, 1.65±0.69 vs. 7.93±1.32 U/ml; GSH-PX, 4.62±1.48 vs. 17.30±2.25 µmol/l; both P<0.01). By contrast, the markers of increased oxidative capacity, MDA and 8-OHdG, were significantly higher in the EM group than in EM-R group (MDA, 4.79±0.32 vs. 1.39±0.32 nmol/ml; 8-OHdG, 24.31±3.48 vs. 5.91±2.68 ng/l; both P<0.001) (Fig. 2). This suggests the presence of oxidative stress, decreased antioxidant capacity and increased oxidative function in the peritoneal cavity of endometriosis mice without rapamycin treatment. Moreover, levels of activation of intraperitoneal oxidative stress were reduced in rapamycin-treated endometriosis mice.

### Rapamycin reduces senescence markers and increases gonadotropin receptors in mouse ovaries

**Figure 3.**
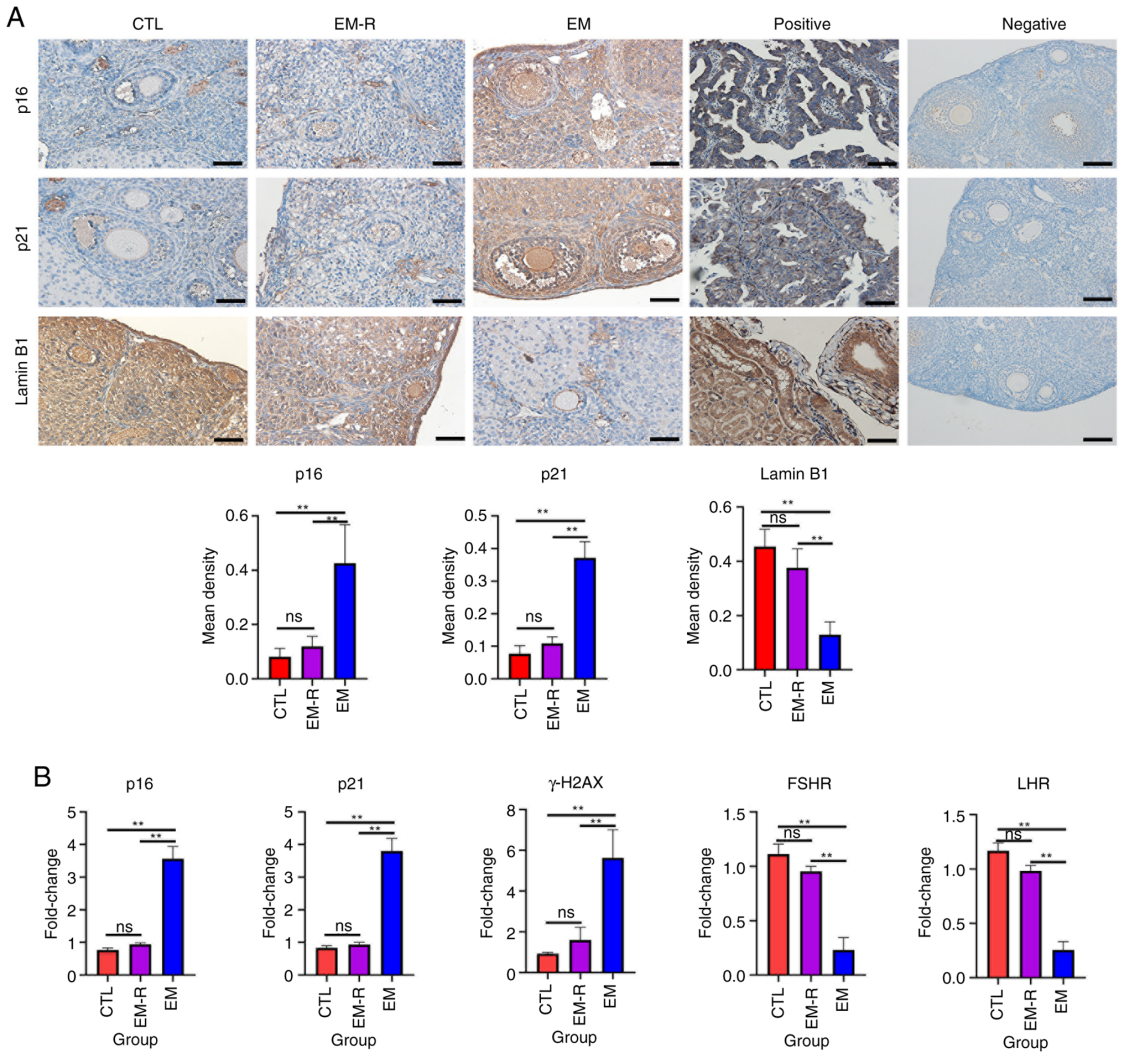
Detection of senescence-related markers and gonadotropin receptor in mice ovarian tissues. (A) Representative immunohistochemical images and the corresponding statistical results of senescence-related markers, p16, p21 and Lamin B1 in the ovarian tissues of three groups, as well as positive and negative controls for immunostaining of p16, p21 and Lamin B1. For positive controls, human endometrial adenocarcinoma tissues were used for p16, human lung tissues were used for p21 and human kidney tissues were used for Lamin B1. p16, p21, Lamin B1 showed positive staining in the nuclei. For negative controls, mouse ovarian tissues were used. The controls all showed negative staining. Scale bar, 50 µm. (B) Gene expression of p16, p21, γH2AX, FSHR and LHR in mice ovarian tissues among the three groups. **P<0.01. FSHR, follicle-stimulating hormone receptor; LHR, luteinizing hormone receptor; CTL, control group; EM-R, endometriosis + rapamycin group; EM, endometriosis group; ns, not statistically significant.

Increased expression of senescence-associated markers, p16, p21 and γH2AX, and decreased expression of Lamin B1 suggest that tissue or cellular senescence is occurring (30). The immunohistochemical experiments in the present study demonstrated that senescence-related markers expression in ovarian granulosa cells and oocyte did not differ significantly between the CTL and EM-R group. However, the expression of senescence-related markers, p16 and p21, in ovarian granulosa cells and oocytes was significantly higher in the EM group than in the EM-R group (p16, 0.42±0.14 vs. 0.12±0.03; p21, 0.37±0.04 vs. 0.11±0.02; both P<0.01). Another senescence-related marker, Lamin B1, was significantly lower in ovarian granulosa cells and oocytes of the EM group compared with the EM-R group (0.13±0.05 vs. 0.37±0.07; P<0.01) (Fig. 3A). This suggests that ovarian tissues in endometriosis mice without rapamycin treatment undergo senescence, and rapamycin could decrease expression of senescence markers in ovaries. Similarly, ovarian tissue PCR experiments revealed significantly higher gene expression of p16, p21 and γH2AX in mice in the EM group than in mice in the EM-R group (p16, 3.57±0.36 vs. 0.94±0.04; p21, 3.80±0.39 vs. 0.93±0.06; γH2AX, 5.63±1.37 vs. 1.61±0.61; all P<0.01). Moreover, gonadotropin receptor (FSHR and LHR) gene expression was significantly higher in the EM-R group than the EM group (FSHR, 0.95±0.04 vs. 0.23±0.11; LHR, 0.98±0.04 vs. 0.25±0.07; both P<0.01) (Fig. 3B).

### Rapamycin improves follicular development

**Figure 4.**
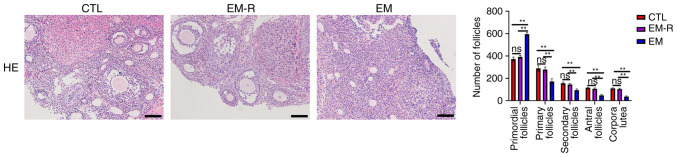
Representative HE staining of ovarian tissues and the number of follicles at each stage in mice of three groups. Scale bar, 100 µm. **P<0.01. CTL, control group; EM-R, endometriosis + rapamycin group; EM, endometriosis group; ns, not statistically significant.

The number of follicles at different stages was not significantly different between the CTL and EM-R groups. However, primordial follicles of the EM group were significantly higher than in the EM-R group (595.6±13.66 vs. 392.1±13.89; P<0.01), whilst primary follicles, secondary follicles, antral follicles and corpus luteum were significantly lower in the EM group than in the EM-R group (primary follicles, 172.2±24.15 vs. 278.6±23.08; secondary follicles, 94.9±10.62 vs. 145.3±9.81; antral follicles, 48±8.56 vs. 106.2±7.88; corpus luteum, 37.8±5.73 vs. 103.6±8.5; All P<0.01). This suggests that endometriosis mice follicles undergo senescence in response to oxidative stress, which leads to impaired development and significant increase in the number of mature follicles in endometriosis mice treated with rapamycin (Fig. 4).

### Activation of PPARα and IGFBP2 in the ovaries of endometriosis mice treated with rapamycin

**Figure 5.**
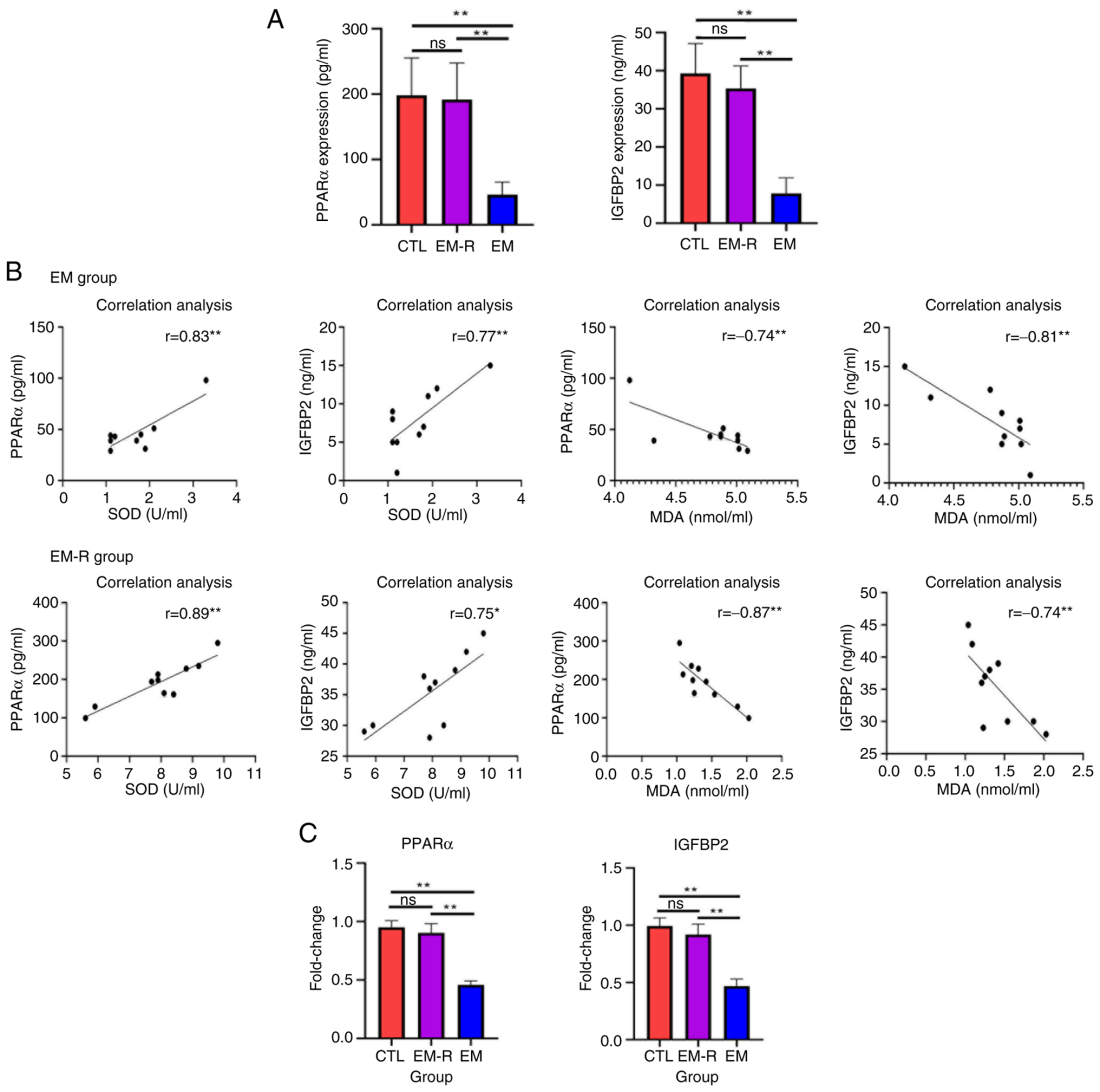
PPARα and IGFBP2 expression in the endometriosis mouse model. (A) Expression of PPARα and IGFBP2 in mouse peritoneal fluid. (B) Correlation between PPARα and IGFBP2 and oxidative stress-related molecules in the EM-R and EM groups. (C) PPARα/IGFBP2 gene expression in mice ovaries of the three groups of mice. *P<0.05; **P<0.01. PPARα, proliferator-activated receptor α; IGFBP2, insulin-like growth factor-binding protein 2; CTL, control group; EM-R, endometriosis + rapamycin group; EM, endometriosis group; ns, not statistically significant; SOD, superoxide dismutase; MDA, malondialdehyde.

The concentration of PPARα and IGFBP2 in the peritoneal fluid of mice in the CTL group were not significantly different from those of the EM-R group; however, the concentration of PPARα and IGFBP2 in the EM group were significantly lower than those in the EM-R group (PPARα, 46.2±19.33 vs. 191.6±56.37 pg/ml; IGFBP2, 7.90±4.04 vs. 35.4±5.89 ng/ml; both P<0.01) (Fig. 5A). Furthermore, the results revealed a positive correlation between SOD and PPARα, and between SOD and IGFBP2, compared with a negative correlation between MDA and PPARα, and between MDA and IGFBP2 in both the EM-R and EM groups (Fig. 5B). In addition, the gene expression of PPARα and IGFBP2 in the ovaries of mice in the EM-R group was not significantly different from that of mice in the CTL group; however, the expression of PPARα and IGFBP2 in the ovaries of mice in the EM group was significantly lower than that of mice in the EM-R group (PPARα, 0.90±0.07 vs. 0.45±0.03; IGFBP2, 0.91±0.09 vs. 0.46±0.06; both P<0.01). This indicated that the expression of PPARα and IGFBP2 in the ovaries of endometriosis mice was significantly decreased, and treatment with rapamycin activated PPARα and IGFBP2 expression (Fig. 5C).

### Rapamycin decreases the expression of senescence markers and increases PPARα and IGFBP2 expression in primary mouse granulosa cells

**Figure 6.**
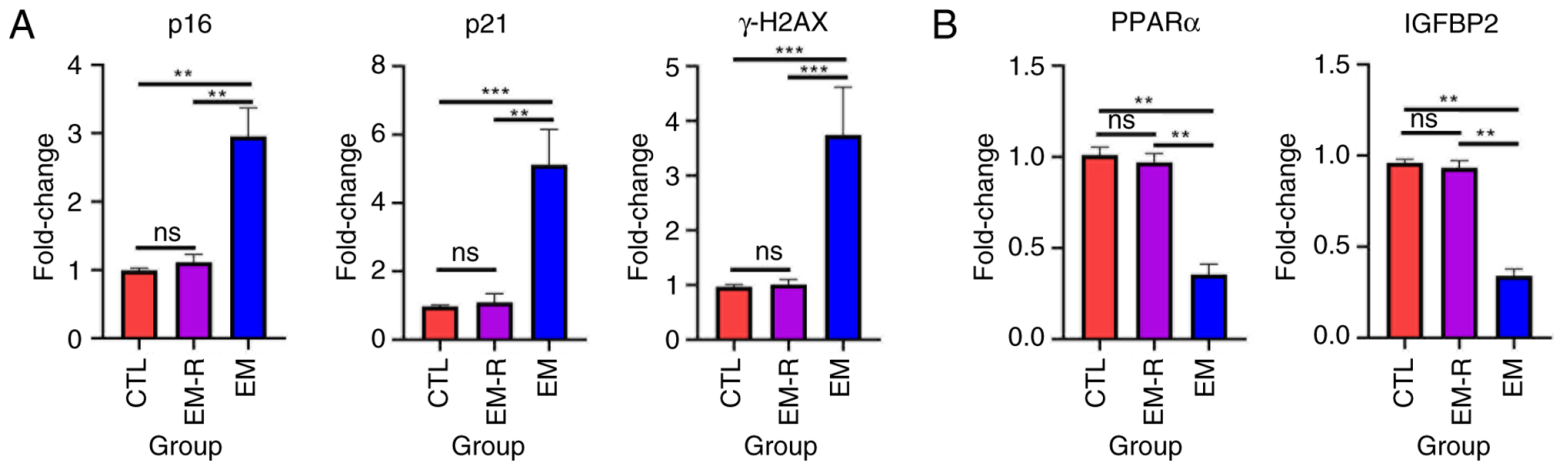
Senescence and PPARα/IGFBP2 expression in primary granulosa cells. Gene expression of (A) p16, p21 and γH2AX and (B) PPARα and IGFBP2 in mice primary granulosa cells among the three groups. **P<0.01; ***P<0.001. PPARα, proliferator-activated receptor α; IGFBP2, insulin-like growth factor-binding protein 2; CTL, control group; EM-R, endometriosis + rapamycin group; EM, endometriosis group; ns, not statistically significant.

Granulosa cells, which closely surround the oocyte, are essential for supporting oocyte development (31). To assess cellular senescence in these cells, primary granulosa cells were isolated from three experimental groups of mice. PCR analysis revealed that granulosa cells from the EM group exhibited significantly elevated levels of senescence-associated markers, including p16, p21 and γH2AX, compared with in the EM-R group (p16, 2.95±0.41 vs. 1.11±0.11; p21, 5.13±1.02 vs. 1.09±0.25; γH2AX, 3.74±0.87 vs. 1.01±0.09; all P<0.01; Fig. 6A). Notably, the expression of these markers in the EM-R group was comparable with that of the CTL group, with no statistically significant differences observed between them (Fig. 6A). By contrast, the expression levels of PPARα and IGFBP2 were significantly reduced in the EM group compared with the EM-R group (PPARα, 0.35±0.55 vs. 0.97±0.04; IGFBP2, 0.34±0.03 vs. 0.93±0.04; both P<0.01; Fig. 6B).

### Rapamycin increases PPARα and IGFBP2 expression and inhibits granulosa cell senescence in vitro

**Figure 7.**
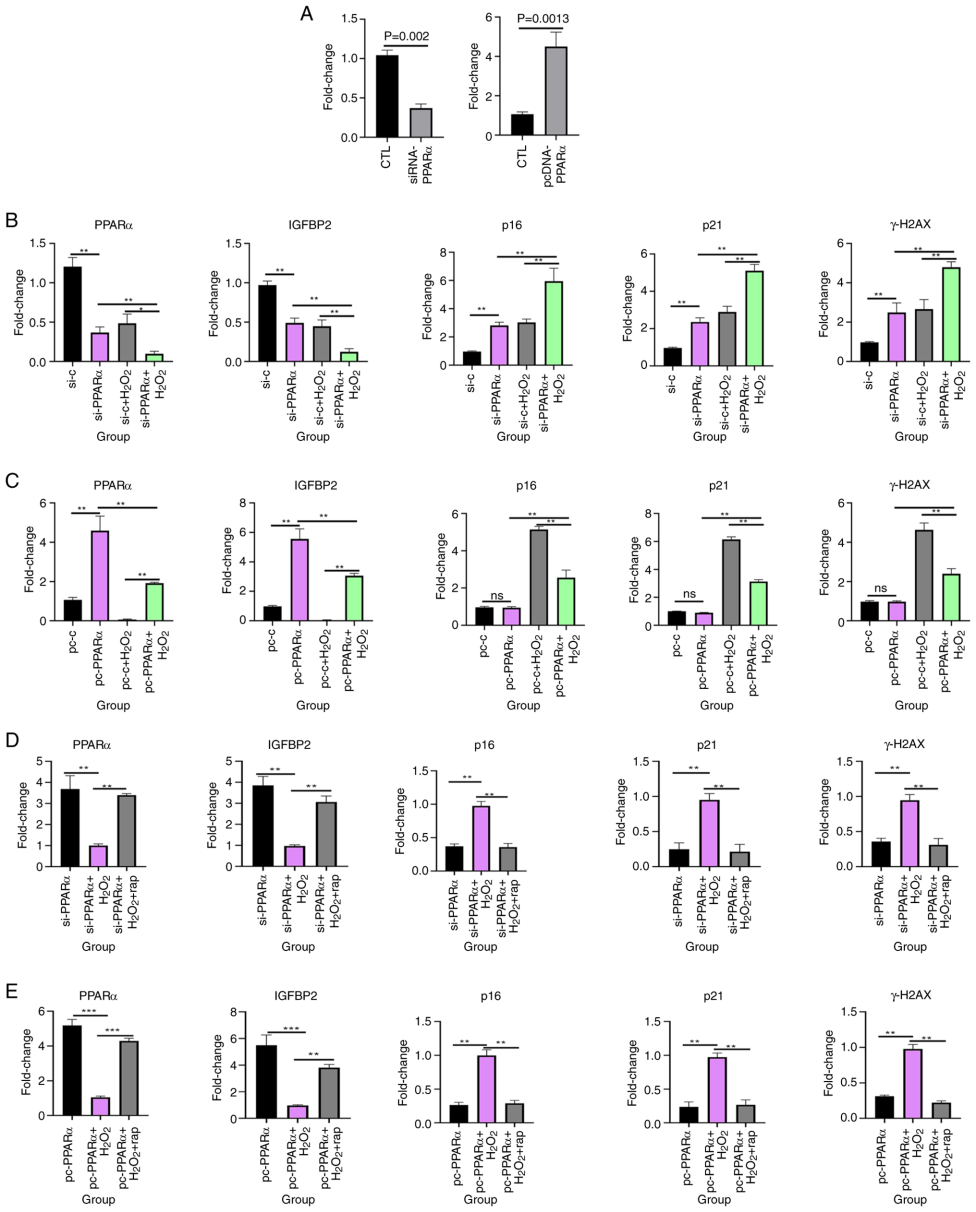
PPARα modulates oxidative stress-induced senescence and IGFBP2 expression in KGN cells. (A) Compared with the blank control group, the PCR results of PPARα after cell transfection with siRNA and overexpression plasmids. Gene expression of PPARα, IGFBP2, p16, p21 and γH2AX in KGN cells treated with H_2_O_2_ after (B) knockdown and (C) overexpression of PPARα. Gene expression of PPARα, IGFBP2, p16, p21 and γH2AX in KGN cells treated with H_2_O_2_ and rapamycin after (D) knockdown and (E) overexpression of PPARα. **P<0.01; ***P<0.001. PPARα, proliferator-activated receptor α; IGFBP2, insulin-like growth factor-binding protein 2; si, small interfering; si-c, non-specific control siRNA; pc, pcDNA; rap, rapamycin; CTL, control; ns, not statistically significant.

To evaluate the functional relationship between PPARα and IGFBP2, knockdown and overexpression of PPARα experiments were performed using KGN cells. First, PCR experiments were performed to assess the expression of PPARα after transfecting cells with siRNA and overexpression plasmids. The results demonstrated that PPARα expression was significantly lower after siRNA transfection (0.37±0.05 vs. 1.04±0.06; P=0.0002) and significantly higher after overexpression plasmid transfection (4.50±0.73 vs. 1.06±0.11; P=0.0013) than in the respective control groups (Fig. 7A). Notably, silencing PPARα significantly reduced IGFBP2 expression, whereas PPARα overexpression significantly increased IGFBP2 levels (Fig. 7B and C). This indicated that IGFBP2 is a downstream target of PPARα.

To assess the role of PPARα in cellular senescence, granulosa cells were treated with H_2_O_2_ following PPARα knockdown or overexpression. The siRNA-PPARα + H_2_O_2_ group exhibited the highest expression of senescence markers (p16, p21 and γH2AX), significantly exceeding levels in the siRNA-control + H_2_O2 group (p16, 5.96±0.90 vs. 3.03±0.23; p21, 5.10±0.34 vs. 2.89±0.30; γH2AX, 4.79±0.27 vs. 2.66±0.48; all P<0.01; Fig. 7B). Conversely, the pcDNA-PPARα + H_2_O_2_ group exhibited lower expression of the senescence markers (p16, p21 and γH2AX) than the pcDNA + H_2_O_2_ group, (p16, 2.56±0.41 vs. 5.13±0.17; p21, 3.13±0.13 vs. 6.15±0.17; γH2AX, 2.40±0.25 vs. 4.63±0.35; all P<0.01; Fig. 7C). PPARα overexpression attenuated H2O2-induced senescence, further supporting the protective role of PPARα against cellular aging. Moreover, cells with knockdown or overexpression of PPARα were treated with H_2_O_2_ for 24 h followed by the addition of 30 µM rapamycin (32) for 48 h, revealed that rapamycin significantly increased PPARα and IGFBP2 expression and significantly decreased the expression of senescence-related markers, p16, p21 and γH2AX. The siRNA-PPARα + H_2_O_2_ group exhibited higher expression of the senescence markers (p16, p21 and γH2AX) than the siRNA-PPARα + H_2_O_2_ + rapamycin group (p16, 0.97±0.06 vs. 0.35±0.05; p21, 0.95±0.09 vs. 0.21±0.10; γH2AX, 0.94±0.08 vs. 0.31±0.09; all P<0.01; Fig. 7D). The pcDNA-PPARα + H_2_O_2_ group exhibited higher expression of the senescence markers (p16, p21 and γH2AX) than the pcDNA-PPARα + H_2_O_2_ + rapamycin group (p16, 0.99±0.08 vs. 0.29±0.04; p21, 0.97±0.06 vs. 0.26±0.07; γH2AX, 0.98±0.06 vs. 0.22±0.02; all P<0.01; Fig. 7E) These findings suggest that rapamycin counteracted senescence by upregulating the PPARα-IGFBP2 axis.

## Discussion

The present study demonstrated that elevated ROS in the peritoneal fluid of endometriosis mice contributed to ovarian senescence and impaired follicular development, whilst rapamycin treatment mitigated these effects by reducing oxidative stress and activating the PPARα/IGFBP2 pathway.

Endometriosis is characterized by a pro-oxidative peritoneal environment (33), with increased ROS levels adversely affecting ovarian function (34,35). It is well documented that oxidative stress is a major source of endogenous and exogenous challenges that promotes senescence and the senescence phenotype (36). A previous study reported that excessive oxidative stress in cumulus granulosa cells triggered cellular senescence which contributed to endometriosis-related infertility (10). Consistent with previous studies (9,10), the present study demonstrated that ROS in peritoneal fluid promoted cellular senescence in ovarian tissues, as shown by elevated senescence-related markers and reduced expression of gonadotropin receptors (FSHR and LHR), critical for follicular maturation (37). This senescence phenotype was associated with a decline in mature follicles and an accumulation of primordial follicles, suggesting impaired folliculogenesis. Rapamycin, an established anti-senescence agent (38), reversed these effects, restoring follicular development and ovarian function.

The mTOR network is an evolutionarily conserved signaling hub that detects and integrates environmental and intracellular nutrients, as well as growth factor signals, thereby orchestrating fundamental cellular and organismal responses such as cell growth, proliferation, apoptosis and inflammation. Previous research supports the notion that mTOR signaling influences lifespan and senescence (39). At present, the only known pharmacological approach to extend lifespan in all studied model organisms, to the best of our knowledge, is the inhibition of the mTOR complex 1 using rapamycin (39). Treatment of hepatocytes with pterostilbene inhibits mTOR and promotes the expression of its downstream molecule, PPARα (40). PPARα serves a crucial role in metabolic homeostasis and aging, with its deficiency associated with fibrotic and senescent phenotypes in several tissues (41). Similarly, IGFBP2 has been implicated in mitigating senescence in pulmonary fibrosis models (42). The findings of the present study revealed for the first time, to the best of our knowledge, that PPARα and IGFBP2 are downregulated in endometriosis-affected ovaries and are negatively associated with senescence. In endometriosis, reduced PPARα/IGFBP2 signaling may exacerbate oxidative stress-induced ovarian damage. Rapamycin restored their expression, suggesting that this pathway is a key mediator in counteracting endometriosis-related ovarian dysfunction.

Whilst the present study characterized folliculogenesis dysfunction in endometriosis-related infertility, it is acknowledged that direct fertility assessments (such as ovulation rates, pregnancy outcomes or litter sizes) were not assessed. These endpoints would provide critical translational insights into how observed follicular abnormalities impact reproductive success. Future studies should integrate longitudinal fertility metrics in murine models, ideally combining hormonal profiling with timed mating trials, to establish functional associations between folliculogenesis defects and infertility phenotypes. Such data would strengthen the clinical relevance of the findings of the present study for patients with endometriosis.

Additionally, although the *in vitro* experiments in the present study demonstrated the regulatory role of the PPARα/IGFBP2 axis in granulosa cells, further *in vivo* validation is warranted to confirm its causal involvement in rapamycin-mediated effects. Pharmacological modulation (such as PPARα agonists/antagonists) or conditional knockout models could elucidate whether targeting this pathway rescues ovarian function in endometriosis. These experiments would not only solidify the mechanistic link, but also explore therapeutic potential, aligning with the broader goal of developing targeted interventions for endometriosis-associated infertility.

In summary, the results of the present study highlight that ROS-induced ovarian senescence contributed to endometriosis-related infertility, and rapamycin counteracted this process by activating the PPARα/IGFBP2 pathway. These findings not only deepen the understanding of endometriosis pathogenesis but also suggest that targeting senescence pathways may offer a promising strategy to improve fertility outcomes in affected women. Future studies should focus on translating these findings into clinical applications.

## Figures and Tables

**Table I.** Names and sequences of all primers used in the present study.

Gene name	Direction	Sequence (5′-3′)
p16 (human)	Forward	AGGCCGATCCAGGTCATGATGA
	Reverse	ACCACCAGCGTGTCCAGGAA
p21 (human)	Forward	GACCTGTCACTGTCTTGTACCCTTG
	Reverse	TTGGAGTGGTAGAAATCTGTCATG
		CTG
FSHR (human)	Forward	TCCCTCCTTGTGCTCAATGTC
	Reverse	GATGTTGGGGTTCCGCACT
LHR (human)	Forward	TTCTGTCTACACCCTCACCG
	Reverse	AAAAGAGCCATCCTCCAAGC
γH2AX (human)	Forward	CAGTGCTGGAGTACCTCAC
	Reverse	GATGATTCGCGTCTTCTTGTTG
PPARα (human)	Forward	TCGGCGAGGATAGTTCTGGAAGC
	Reverse	ACCACAGGATAAGTCACCGAGGAG
IGFBP2 (human)	Forward	ACAGTGCAAGATGTCTCTGAACGG
	Reverse	GCCTCCTGCTGCTCATTGTAGAAG
GAPDH (human)	Forward	GCACCGTCAAGGCTGAGAAC
	Reverse	TGGTGAAGACGCCAGTGGA
p16 (mouse)	Forward	CGCAGGTTCTTGGTCACTGT
	Reverse	TGTTCACGAAAGCCAGAGCG
p21(mouse)	Forward	CCTGGTGATGTCCGACCTG
	Reverse	CCATGAGCGCATCGCAATC
FSHR (mouse)	Forward	CCTTGCTCCTGGTCTCCTTG
	Reverse	CTCGGTCACCTTGCTATCTTG
LHR (mouse)	Forward	CAGCTGCCTTCAAAGTACCC
	Reverse	TTGGCACAAGAATTGACAGG
γH2AX (mouse)	Forward	GGTGCTCGAGTACCTCACTG
	Reverse	CTTGTTGAGCTCCTCGTCGT
PPARα (mouse)	Forward	CAAGGCCTCAGGGTACCACT
	Reverse	TTGCAGCTCCGATCACACTT
IGFBP2 (mouse)	Forward	CCTTGCCAGCAGGAGTTG
	Reverse	TCCGTTCAGAGACATCTTGC
GAPDH (mouse)	Forward	GGTTGTCTCCTGCGACTTCA
	Reverse	TGGTCCAGGGTTTCTTACTCC

FSHR, follicle-stimulating hormone receptor; LHR, luteinizing hormone receptor; PPARα, proliferator-activated receptor α; IGFBP2, insulin-like growth factor-binding protein 2.

## Data Availability

The data generated in the present study may be requested from the corresponding author.
